# Comparative analysis of right ventricular metabolic reprogramming in pre-clinical rat models of severe pulmonary hypertension-induced right ventricular failure

**DOI:** 10.3389/fcvm.2022.935423

**Published:** 2022-09-09

**Authors:** Somanshu Banerjee, Jason Hong, Soban Umar

**Affiliations:** ^1^Division of Molecular Medicine, Department of Anesthesiology and Perioperative Medicine, Los Angeles, CA, United States; ^2^Division of Pulmonary and Critical Care Medicine, Department of Medicine, David Geffen School of Medicine, University of California Los Angeles (UCLA), Los Angeles, CA, United States

**Keywords:** pulmonary hypertension, right ventricular failure, targeted metabolomics, monocrotaline, Joint Pathway Analysis, multi-omics, Sugen/Hypoxia

## Abstract

**Background:**

Pulmonary hypertension (PH) leads to right ventricular (RV) hypertrophy and failure (RVF). The precise mechanisms of the metabolic basis of maladaptive PH-induced RVF (PH-RVF) are yet to be fully elucidated. Here we performed a comparative analysis of RV-metabolic reprogramming in MCT and Su/Hx rat models of severe PH-RVF using targeted metabolomics and multi-omics.

**Methods:**

Male Sprague Dawley rats (250–300 gm; *n* = 15) were used. Rats received subcutaneous monocrotaline (60 mg/kg; MCT; *n* = 5) and followed for ~30-days or Sugen (20 mg/kg; Su/Hx; *n* = 5) followed by hypoxia (10% O_2_; 3-weeks) and normoxia (2-weeks). Controls received saline (Control; *n* = 5). Serial echocardiography was performed to assess cardiopulmonary hemodynamics. Terminal RV-catheterization was performed to assess PH. Targeted metabolomics was performed on RV tissue using UPLC-MS. RV multi-omics analysis was performed integrating metabolomic and transcriptomic datasets using Joint Pathway Analysis (JPA).

**Results:**

MCT and Su/Hx rats developed severe PH, RV-hypertrophy and decompensated RVF. Targeted metabolomics of RV of MCT and Su/Hx rats detected 126 and 125 metabolites, respectively. There were 28 and 24 metabolites significantly altered in RV of MCT and Su/Hx rats, respectively, including 11 common metabolites. Common significantly upregulated metabolites included aspartate and GSH, whereas downregulated metabolites included phosphate, α-ketoglutarate, inositol, glutamine, 5-Oxoproline, hexose phosphate, creatine, pantothenic acid and acetylcarnitine. JPA highlighted common genes and metabolites from key pathways such as glycolysis, fatty acid metabolism, oxidative phosphorylation, TCA cycle, etc.

**Conclusions:**

Comparative analysis of metabolic reprogramming of RV from MCT and Su/Hx rats reveals common and distinct metabolic signatures which may serve as RV-specific novel therapeutic targets for PH-RVF.

## Introduction

Pulmonary hypertension (PH) is a chronic, progressive, and fatal pulmonary vascular disease that leads to increased right ventricular hypertrophy (RVH), RV failure (RVF), and ultimately, death (1–7). With about 200,000 Americans hospitalized each year with PH, the estimated prevalence for PH is between 15 and 50 cases per million individuals. If left untreated, the life span of an individual with PH is about 2.8 years and the 5-year survival rate is only around 62% (8). PH-induced RV failure (PH-RVF) is a major determinant of morbidity and mortality in PH (9–11) and is characterized by RV myocyte hypertrophy (12–14), extensive extra-cellular matrix (ECM) reorganization (14–16), fibrosis (17–21) and vascular remodeling (22–24).

In the setting of chronic pressure-overload associated with PH, the process of RV remodeling is continuous but often dichotomized into adaptive or compensated and maladaptive or decompensated phenotypes. RVH is initially adaptive, but can eventually lead to RV decompensation, dilatation, and failure (9, 25, 26). Compensated RV remodeling is typically associated with normal RV function and is characterized by concentric hypertrophy, minimal RV dilatation and fibrosis (9, 25). On the other hand, decompensated RV remodeling is defined by reduced RV function and is characterized by extensive inflammation and fibrosis, oxidative stress, capillary rarefaction, myocyte apoptosis, metabolic reprogramming, and glycolytic shift (9, 25, 26).

RV appears to exhibit a distinct stepwise metabolic reprogramming, depending upon the transition from normal to adaptive and maladaptive remodeling from chronic pressure overload due to pulmonary vascular remodeling (27). While transitioning from the compensated to the decompensated state, RV cardiomyocytes experience a metabolic shift including: (1) reduced oxidative phosphorylation and beta-oxidation of fatty acids (24, 27, 28); (2) pyruvate to lactate conversion through aerobic glycolysis (Warburg effect) and its utilization (27, 28); and (3) increased utilization of amino acids especially glutamine (glutaminolysis) in the TCA cycle (27–29). Despite some prior reports (30–32) on metabolic reprogramming of RV in experimental PH, a comparative analysis of RV metabolome using targeted metabolomics and multi-omics approaches in the pre-clinical MCT and Su/Hx rat models of severe decompensated PH-RVF is missing. Hence, there is an unmet need to further elucidate the RV-specific metabolic therapeutic targets to devise potentially novel RV-specific therapeutic strategies based on metabolite supplementation and/or pathway-specific gene manipulation.

Recently we demonstrated significantly similar RV transcriptomic signatures between MCT and Su/Hx rat models of severe PH-RVF (24). We found that fatty acid metabolism and oxidative phosphorylation (OXPHOS), two critically important metabolic pathways tightly associated with cardiomyocyte mitochondrial bioenergetics, contractility, and functioning, were the top common downregulated pathways in both rat models (24). Hence, here we hypothesized that these two rat models may also exhibit similar RV metabolomic signatures. Therefore, we performed a comprehensive comparative targeted metabolomics analysis on RV tissue of MCT and Su/Hx rats, that recapitulate most of the pathophysiology of human PH-RVF. Further, we performed RV multi-omics analysis integrating metabolomic and transcriptomic (24) datasets using Joint Pathway Analysis (JPA).

## Materials and methods

### Animals and treatments

**Figure 1:**
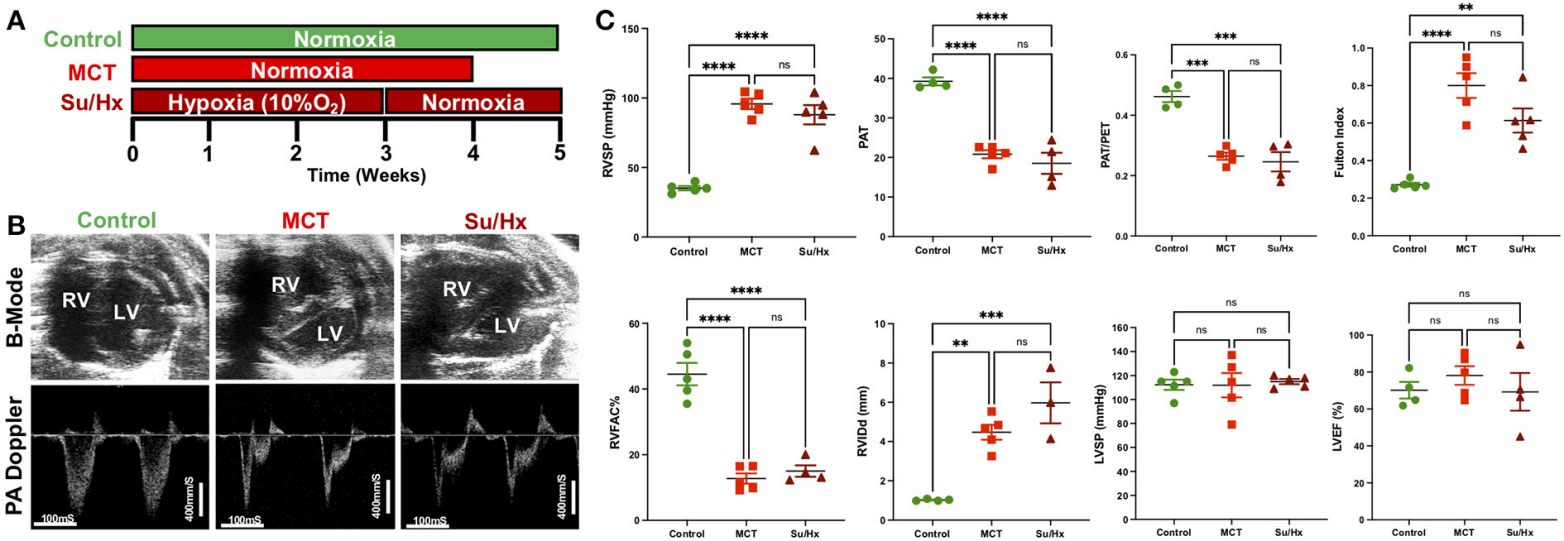
Development of severe decompensated RV Failure in MCT and Su/Hx rats. **(A)** Experimental protocol. **(B)** Representative transthoracic echocardiographic images of B-Mode (upper panel) of the heart in parasternal short axis view at end diastole and pulmonary artery (PA) pulsed-wave doppler (lower panel) images from Control, MCT and Su/Hx rats. **(C)** Plots comparing RVSP (mmHg), PAT (mS), PAT/PET, Fulton Index [RV/(LV + IVS)], RVFAC (%), RVIDd (mm), LVSP (mmHg) and LVEF (%) in Control, MCT and Su/Hx rats. Data presented as mean ± SEM. *N* = 3–5 per group. **p < 0.01, ***p < 0.001, ****p < 0.0001; ns, not significant.

All animal studies were performed in accordance with the National Institutes of Health (NIH) Guide for the Care and Use of Laboratory Animals. Adult male Sprague Dawley rats (200–250 g) received either a single subcutaneous injection of endothelial toxin Monocrotaline (MCT, 60 mg/kg, MCT group, *n* = 5) and were followed for ~30 days or VEGF-receptor antagonist Sugen (SU5416, 20 mg/kg, Su/Hx group, *n* = 5) and kept in hypoxia (10% oxygen) for 3-weeks followed by 2-weeks of normoxia. PBS treated rats served as controls (CTRL group, *n* = 5) (Figure 1A).

### Echocardiography and RV catheterization

Transthoracic echocardiogram (VisualSonics Vevo2100) was obtained using a rat specific probe (30 MHz). Rats were anesthetized *via* inhaled isoflurane at 2–3%. Each rat was placed in supine position, and body temperature was maintained at 37°C. Transthoracic echocardiography was performed to monitor cardiopulmonary hemodynamics using a Vevo 2,100 high-resolution image system (VisualSonics, Toronto, Canada). Echocardiograms including B-mode, M-mode and pulsed wave Doppler images were obtained (Figure 1B). RV fractional area of change (RVFAC, %) was measured from parasternal short-axis view at mid-papillary level. RV internal dimension at end-diastole (RVIDd, mm) was measured using M-mode, parasternal short or long-axis view. A 30-MHz linear transducer was used to perform the pulmonary pulsed-wave Doppler echocardiography of PA flow (Figure 1B). The probe was placed in a parasternal long-axis position to visualize the PA outflow tract. Pulsed flow Doppler imaging was then overlaid to observe the dynamics of blood flow through the PA valve and measure pulmonary ejection time (PET) and pulmonary acceleration time (PAT). Ejection fraction (EF) was measured from left ventricle. Echocardiogram software (Vevo 2,100 version: 1.5.0) was used for all echocardiography measurements.

The right ventricular systolic pressure (RVSP) and left ventricular systolic pressure (LVSP) were measured directly by inserting a catheter (1.4 F Millar SPR-671, ADInstruments) connected to a pressure transducer (Power Lab, ADInstruments) into the RV or LV, respectively, just before sacrifice. Briefly, for cardiac catheterization, the rats were anesthetized with isoflurane (2–3%). The animals were placed on a controlled warming pad to keep the body temperature constant at 37°C. After a tracheotomy was performed, a cannula was inserted, and the animals were mechanically ventilated. After a midsternal thoracotomy, rats were placed under a stereomicroscope (Zeiss, Hamburg, Germany) and a pressure-conductance catheter (model 1.4 F Millar SPR-671) was introduced *via* the apex into the RV or LV and positioned toward the pulmonary or aortic valve, respectively. The catheter was connected to a signal processor (ADInstruments) and pressures were recorded digitally. After recording the pressures, heart and lung tissues were removed rapidly under deep anesthesia for preservation of protein and RNA integrity.

### Gross histologic analysis, tissue preparation, and imaging

The right ventricular (RV) wall, the left ventricular (LV) wall, and the interventricular septum (IVS) were dissected. RV, LV, IVS and lungs were weighed. The ratio of the RV to LV plus septal weight [RV/(LV + IVS)] was calculated as the Fulton index of RV hypertrophy. RV free wall tissue was quickly washed in ice-cold PBS and immediately snap frozen in liquid nitrogen for subsequent metabolomics analysis.

Lungs were frozen, and transversal 5 μm sections were obtained with a cryostat. Lung tissue sections were stained with Masson's trichrome according to the manufacturer's protocol, and images were acquired with a confocal microscope (Nikon). For assessment of pulmonary arteriolar wall thickness, only distal pulmonary arteries <100 μm were quantified using ImageJ software.

### Targeted metabolomics of RV tissue

Snap frozen RV tissue was pulverized in liquid nitrogen (Liq.N_2_) using mortar and pestle. Protein estimation was performed using Bradford assay method (33) and normalized RV samples were processed for metabolite extraction. Targeted metabolomics approach was applied for detection and quantification of a set of 150 central carbon metabolites.

### Metabolite extraction from RV tissue

Briefly, 50 mg of RV tissue was pulverized using Liq. N_2_ in mortar and pestle and immediately put in pre-chilled 1.5 ml microcentrifuge tubes. Next, 1 ml 80% methanol (MeOH, pre-chilled at −80°C) was added to each sample and vortexed vigorously for 20 s to resuspend well. The samples were then transferred to 2 ml microcentrifuge tubes with 0.7 ml 80% MeOH and kept for 3 h at −80°C to aid proper quenching, extraction, and protein precipitation. Then the samples were vortexed again for 20 s and centrifuged at 16,000 g for 15 min @ 4°C. The entire supernatant was transferred to a new 2 ml microcentrifuge tube, added 80% MeOH to reach the final volume to 200 uL, dried using the Genevac EZ-2Elite evaporator at 30°C using program 3 (aqueous) and the tubes with the remaining pellets were stored at −80°C. The dried samples were stored at −80°C until ready for LC-MS analysis. LC-MS analysis was performed using Thermo Scientific Q Exactive mass spectrometers coupled to UltiMate 3,000 UPLC chromatography systems at the UCLA Metabolomics Core Facility (34).

### Bioinformatics analysis

The targeted metabolomics raw data was processed for further analysis using MetaboAnalyst 5.0 for metabolomic pathway enrichment analysis. Further, Joint Pathway Analysis (JPA) was performed by integrating the transcriptomic data from our recently published study (24) and the metabolomic data from our current study using MetaboAnalyst 5.0 to correlate the genes and metabolites related to RV metabolic reprogramming in MCT and Su/Hx rats from two separate sets of experiments (35, 36). For pathway analysis, an FDR adjustment (<0.05) was performed.

### Validation cohort of targeted metabolomics of RV tissue from MCT and Su/Hx rats

A validation cohort of targeted metabolomics of RV tissue was performed on a separate set of male Sprague Dawley rats with comparable disease severity (Control *n* = 4, MCT *n* = 4, Su/Hx *n* = 3).

### Statistical analysis

A sample size of 5 rats per group gives us adequate power (80%) to detect standardized effect sizes between groups as small as 2.5 (assuming a two-sample *t*-test, Bonferroni adjusted alpha = 0.017, two-tailed). To assess differences between groups, Welch's Unpaired *t*-test and one-way ANOVA tests were used due to potential assumption violations (equal variance) using the more standard tests. When significant differences were detected, individual mean values were compared by *post-hoc* tests that allowed for multiple comparisons with adequate type I error control (Tukey's). Analyses were run using GraphPad Prism 9.0 and *p*-values < 0.05 was considered statistically significant. Values are expressed as mean ± SEM.

## Results

### Development of RVF in MCT and Su/Hx rats

Both MCT and Su/Hx rats showed severe PH as evidenced by increased RVSP (Control: 35.1 ± 1.5 mmHg, MCT: 95.8 ± 3.7 mmHg, *p* < 0.0001 vs. Control; Su/Hx: 87.9 ± 6.9 mmHg, *p* < 0.0001 vs. Control), and decreased pulmonary artery acceleration time (PAT) (Control: 39.3 ± 1.0 mS, MCT: 20.8 ± 1.0 mS, *p* < 0.0001 vs. Control; Su/Hx: 18.5 ± 2.7 mS, *p* < 0.0001 vs. Control) and PAT/pulmonary ejection time (PET) ratio (Control: 0.46 ± 0.01, MCT: 0.26 ± 0.01, *p* = 0.0001 vs. Control; Su/Hx: 0.25 ± 0.03, *p* = 0.0001 vs. Control) compared to control (Figure 1C). Both MCT and Su/Hx rats showed severe pulmonary vascular remodeling as the hallmark of PH. Significantly increased pulmonary arteriolar medial hypertrophy was documented in both MCT and Su/Hx rats (% wall thickness: Control: 14.2 ± 1.0, MCT: 43.7 ± 2.4, *p* < 0.0001 vs. Control; Su/Hx: 45.8 ± 3.7, *p* < 0.0001 vs. Control; Supplementary Figure 1). MCT and Su/Hx rats also demonstrated an increase in RV hypertrophy Fulton index (RV/LV + IVS) (Control: 0.27 ± 0.01, MCT: 0.80 ± 0.06, *p* < 0.0001 vs. Control; Su/Hx: 0.61 ± 0.06, *p* = 0.0019 vs. Control). Decompensated RV failure was demonstrated by decreased RV fractional area change (RVFAC) in MCT and Su/Hx rats (Control: 44.5 ± 3.4%, MCT: 12.7 ± 1.5%, *p* < 0.0001 vs. Control; Su/Hx: 15.0 ± 1.7%, *p* < 0.0001 vs. Control) and increased RV internal diameter at end-diastole (RVIDd) (Control: 1.0 ± 0.02 mm, MCT: 4.4 ± 0.38 mm, *p* = 0.0019 vs. Control; Su/Hx: 5.9 ± 1.04 mm, *p* = 0.0004 vs. Control) compared to control (Figure 1C). No significant differences were observed between Su/Hx- and MCT-treated groups for all parameters. No significant differences were observed in Su/Hx- and MCT-treated groups compared to control for LVSP (Control: 112.3 ± 4.3 mmHg, MCT: 111.9 ± 10.1 mmHg, *p* = 0.9991 vs. Control; Su/Hx: 115.0 ± 2.1 mmHg, *p* < 0.9528 vs. Control) and LVEF (Control: 70.1 ± 4.5%, MCT: 78.1 ± 5.0 %, *p* = 0.6862 vs. Control; Su/Hx: 69.3 ± 10.2 %, *p* = 0.9959 vs. Control) (Figure 1C).

### Targeted metabolomics of the RV in MCT rats

**Figure 2:**
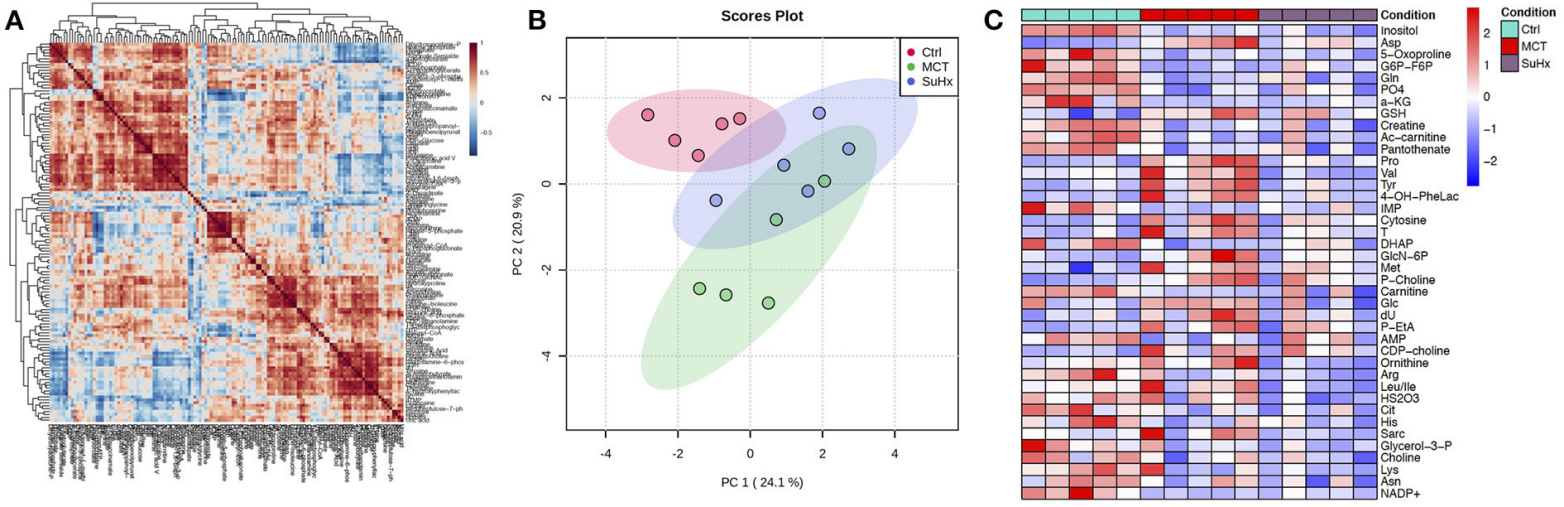
Comparative analysis of targeted metabolomics of RV tissue from severe decompensated RV Failure in MCT and Su/Hx rats. **(A)** Correlation heat map of individual metabolites from targeted metabolomics data of RV tissue from Control, MCT and Su/Hx rats. Red color represents positive correlation and blue color represents negative correlation. **(B)** Scores Plot showing PC1 plotted against PC2 for individual data points of Control (red), MCT (green) and Su/Hx (blue) rats. **(C)** Heat map showing scaled expression of 43 differentially expressed metabolites from RV tissue of Control (blue), MCT (red) and Su/Hx (purple) rats. *N* = 5 per group.

**Figure 3:**
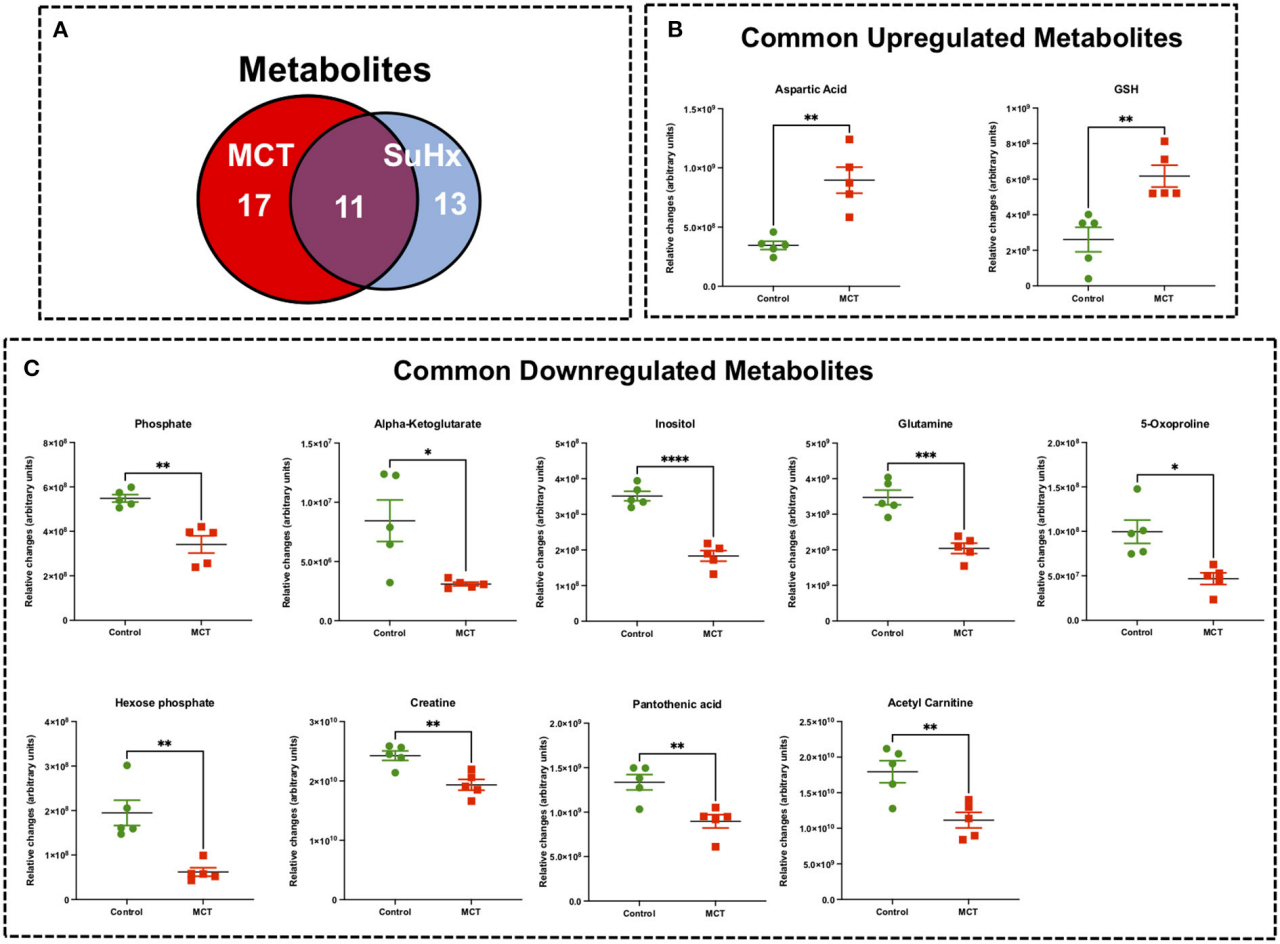
Significant differentially expressed metabolites from RV tissue of MCT rats that are also significantly altered in Su/Hx rat RV tissue. **(A)** Venn diagram showing significantly regulated metabolites in MCT vs. Control (red) and Su/Hx vs. Control (blue). **(B)** Significantly upregulated metabolites from RV tissue of MCT rats (red) that are common with Su/Hx rat RV tissue compared to Control rats (green). **(C)** Significantly downregulated metabolites from RV tissue of MCT rats (red) that are common with Su/Hx rat RV tissue compared to Control rats (green). Data presented as mean ± SEM. *N* = 5 per group. *p < 0.05, **p < 0.01, ***p < 0.001, ****p < 0.0001.

**Figure 4:**
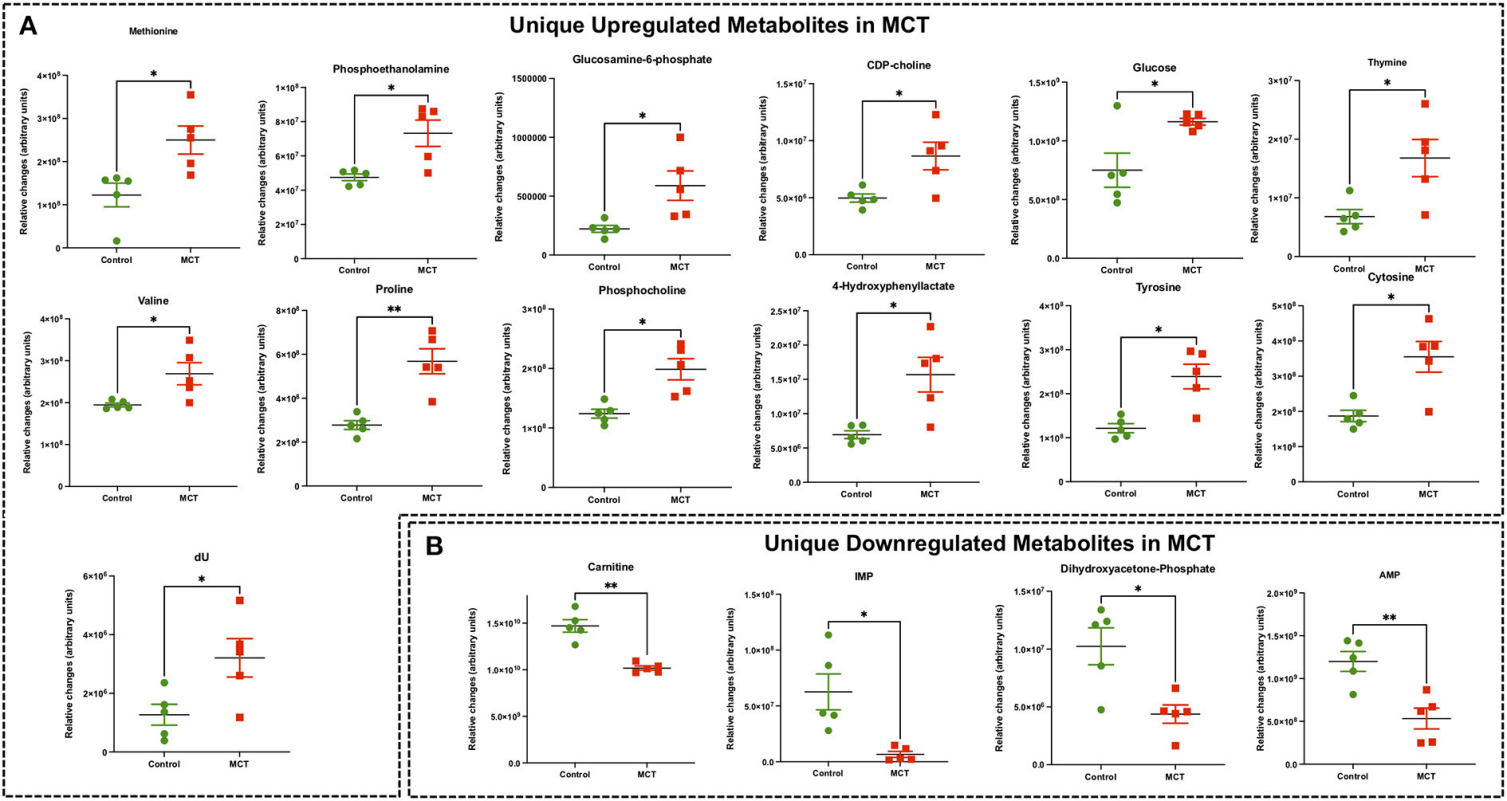
Significant differentially expressed metabolites from RV tissue unique to MCT rats. **(A)** Significantly upregulated metabolites from RV tissue unique to MCT rats (red) with severe decompensated RV Failure compared to Control rats (green). **(B)** Significantly downregulated metabolites from RV tissue unique to MCT rats (red) with severe decompensated RV Failure compared to Control rats (green). Data presented as mean±SEM. *N* = 5 per group. *p < 0.05, **p < 0.01.

Targeted metabolomics of the RV free wall tissue of MCT rats detected 126 metabolites (Figures 2, 3, Supplementary Figures 2–6). There were 28 metabolites significantly altered in RV of MCT rats compared to controls (*p* < 0.05). Out of these 28 metabolites, 15 were upregulated and 13 were downregulated. Based on *p*-value, the top 10 significantly altered metabolites in MCT vs. control were inositol, carnitine, glutamine, phosphate, proline, aspartic acid, GSH, hexose phosphate and creatine. The top 5 significantly upregulated metabolites included proline, aspartic acid, tyrosine, GSH and phosphocholine. The top 5 significantly downregulated metabolites included inositol, carnitine, glutamine, phosphate, and hexose phosphate (Figures 3, 4).

### Targeted metabolomics of the RV in Su/Hx rats

**Figure 5:**
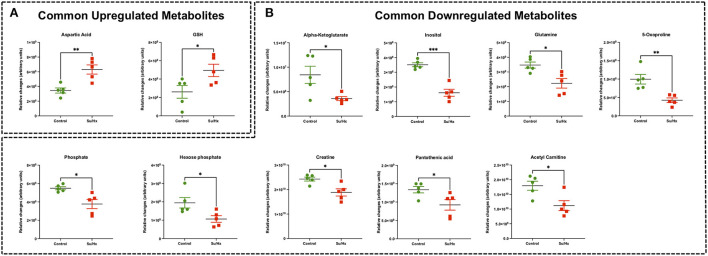
Significant differentially expressed metabolites from RV tissue of Su/Hx rats that are also significantly altered in MCT rat RV tissue. **(A)** Significantly upregulated metabolites from RV tissue of Su/Hx rats (red) that are common with MCT rat RV tissue compared to Control rats (green). **(B)** Significantly downregulated metabolites from RV tissue of Su/Hx rats (red) that are common with Su/Hx rat RV tissue compared to Control rats (green). Data presented as mean ± SEM. *N* = 5 per group. *p < 0.05, **p < 0.01, ***p < 0.001.

**Figure 6:**
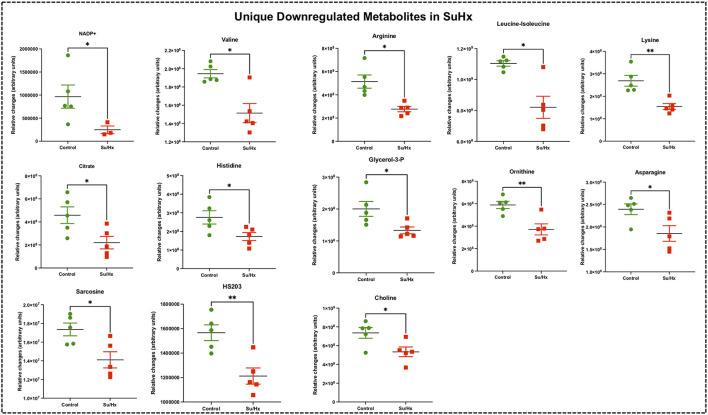
Significant differentially expressed metabolites from RV tissue unique to Su/Hx rats. Significantly expressed metabolites from RV tissue unique to Su/Hx rats (red) with severe decompensated RV Failure compared to Control rats (green). Data presented as mean ± SEM. *N* = 5 per group. *p < 0.05.

Targeted metabolomics of the RV free wall tissue of Su/Hx rats detected 125 metabolites (Figures 2, 3, Supplementary Figures 2–6). There were 24 metabolites significantly altered in RV of Su/Hx rats compared to controls (*p* < 0.05). Out of these 24 metabolites, 2 were upregulated and 22 were down regulated. Based on *p*-value, the top 10 significantly altered metabolites in Su/Hx vs. control were inositol, lysine, aspartic acid, 5-Oxoproline, succinate, arginine, leucine-isoleucine, thiosulfate, valine, and ornithine. The top 2 significantly upregulated metabolites included aspartic acid and GSH. The top 5 significantly downregulated metabolites included inositol, lysine, 5-Oxoproline, succinate, and arginine (Figures 5, 6).

### Common RV metabolomic signature of MCT and Su/Hx rats

There were 11 common significantly altered RV metabolites between MCT and Su/Hx rats (Figure 3, Supplementary Figure 7). There were 2 common upregulated and 9 common downregulated metabolites. The common significantly upregulated metabolites included aspartic acid and GSH. The common significantly downregulated metabolites included inositol, glutamine, creatine, phosphate, hexose phosphate, α-ketoglutarate, pantothenic acid, acetylcarnitine and 5-Oxoproline. Interestingly, valine was upregulated in MCT but downregulated in Su/Hx (Figures 3, 5).

### RV metabolic pathway analysis highlights common metabolic reprogramming signature

**Figure 7:**
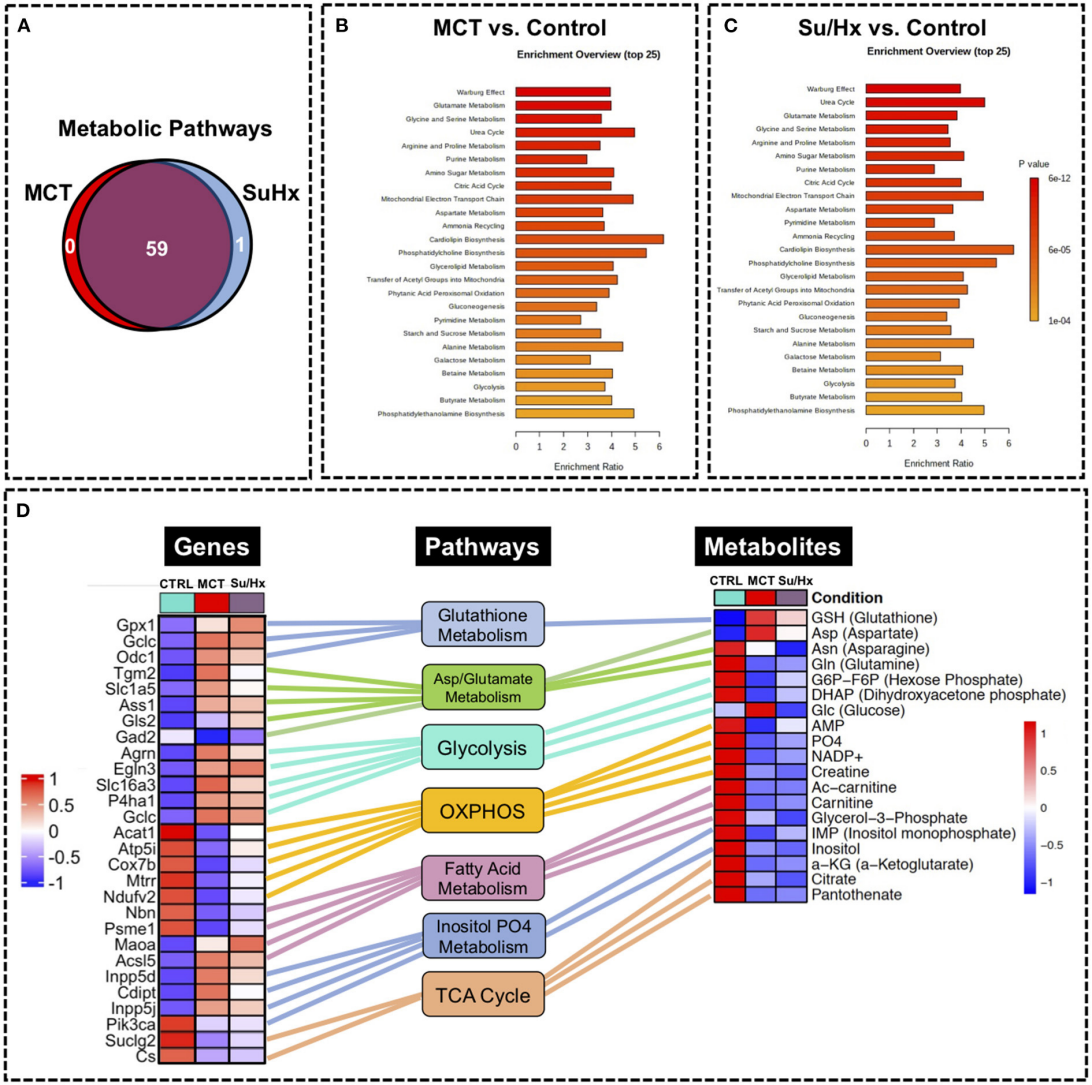
Metabolic pathway enrichment analysis showing RV metabolic reprogramming and Joint Pathway Analysis (JPA) of transcriptomic and metabolomic datasets from RV tissue of MCT and Su/Hx rats highlighting the top common genes, metabolites and metabolic pathways. **(A)** Venn diagram showing significantly regulated pathways in MCT vs. Control (red) and Su/Hx vs. Control (blue). **(B,C)** Metabolomic Pathway Enrichment Analysis showing 25 top significantly regulated metabolic pathways in RV tissue of MCT vs. Control and Su/Hx vs. Control rats based on enrichment ratio and FDR <0.05. **(D)** For integrative analysis of transcriptomics (24) and metabolomics data at the pathway level, list of significant genes (FDR <0.05) from the transcriptomic data with official gene names and fold changes and list of metabolites from metabolomics data with compound names and fold changes, were uploaded in JPA module of Metaboanalyst 5.0 platform and analyzed for all pathways (integrated) by selecting algorithms: (1) Hypergeometric test for Enrichment analysis, (2) Degree centrality for Topology measure and (3) Combined *p*-values (pathway level) for the integration method. Here, only the critical metabolic pathways that are typically associated with cardiomyocyte hypertrophy and contractile function such as glutathione metabolism, aspartate and glutamate metabolism, glycolysis, oxidative phosphorylation, fatty acid metabolism, inositol metabolism and TCA (citric acid) cycle are shown. In the top common significant genes (left heat map; 28 genes from 8 pathways) and metabolites (right heat map; 19 metabolites from 8 pathways), corresponding to each of the above-mentioned pathways (middle panel, rectangular boxes), are represented with their normalized differential expression and normalized average amounts [top upregulated (red) and top downregulated (blue) (FDR <0.05)], respectively. Colored connecting lines (color similar to the metabolic pathway box) between genes and pathways as well as metabolites and pathways, are drawn to show the association of genes and metabolites with their corresponding metabolic pathways.

We performed metabolic pathway enrichment analysis using RV metabolomics data which highlighted 59 significantly altered pathways in MCT and 60 significantly altered pathways in Su/Hx (based on FDR < 0.05) (Figure 7A). Importantly, there was significant concordance between MCT and Su/Hx as demonstrated by 59 common significantly altered pathways shared between the two models. Interestingly, Warburg effect was the top common metabolic pathway between MCT and Su/Hx. Other top common metabolic pathways included glutamate metabolism, glycine and serine metabolism, arginine and proline metabolism, aspartate metabolism, citric acid (TCA) cycle, mitochondrial electron transport chain, glycolysis, gluconeogenesis, and several others (Figures 7B,C).

### Joint Pathway Analysis using transcriptomic and metabolomic datasets

We performed JPA on RV transcriptomic dataset from our recently published study (24) and metabolomic dataset from the current study (Figure 7D). JPA highlighted common genes and metabolites related to key metabolic pathways such as glutathione metabolism, aspartate and glutamate metabolism, glycolysis, oxidative phosphorylation, fatty acid metabolism, inositol metabolism and TCA (citric acid) cycle among others (Figure 7D). Some of these genes (Cox7b, Acat1) have already been validated by PCR (24).

### Confirmation of RV targeted metabolomics using a validation cohort

We ran a validation cohort of targeted metabolomics of RV tissue on a separate set of rats with comparable disease severity (Control *n* = 4, MCT *n* = 4, Su/Hx *n* = 3) and found very similar results to the original cohort (Supplementary Figure 8).

## Discussion

**Figure 8:**
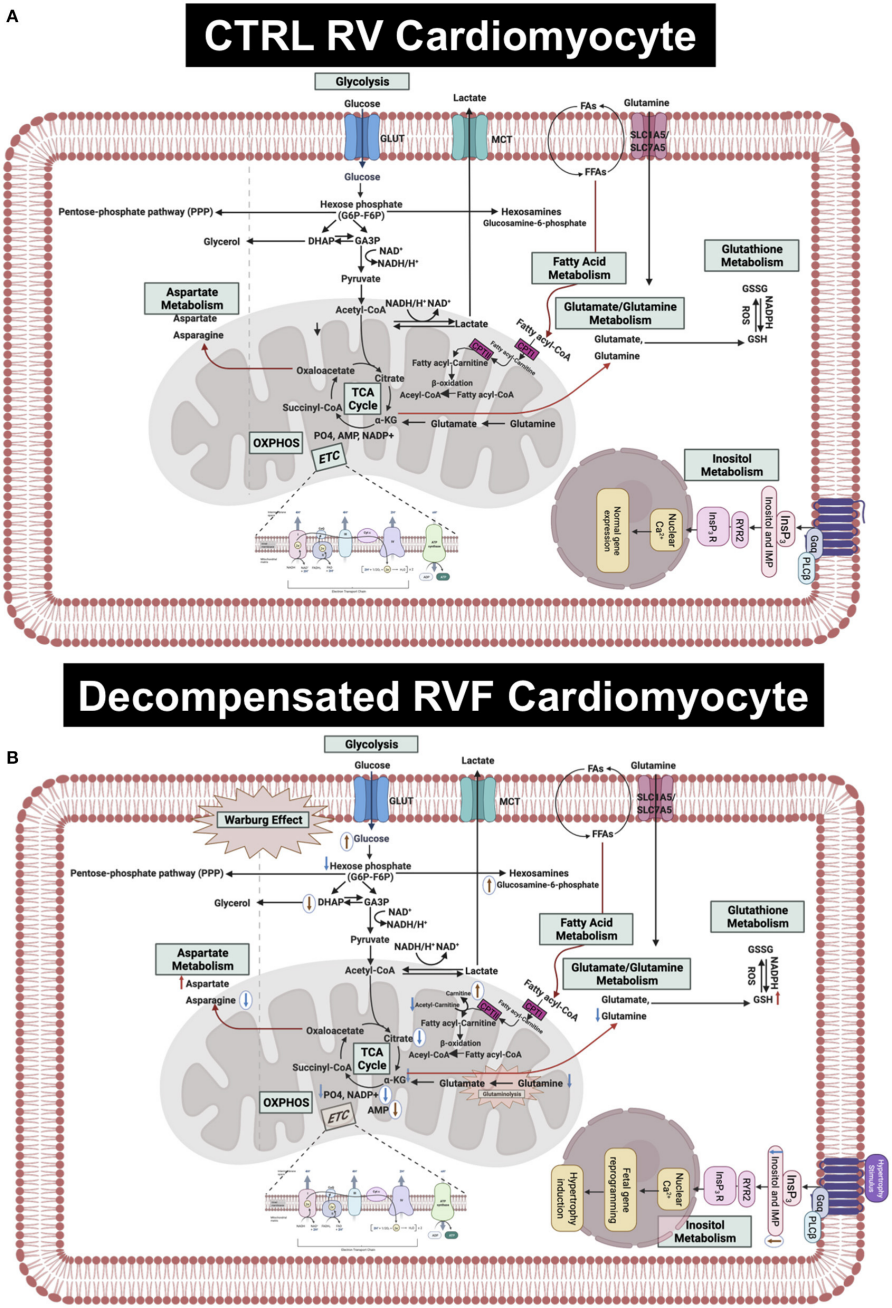
Hypothetical scheme summarizing the results of this study. **(A)** Upper panel shows the normal metabolism in RV cardiomyocytes in CTRL rats. **(B)** Lower panel shows the altered metabolites and metabolic pathways in the decompensated RVF cardiomyocytes of MCT and Su/Hx rats. The common significantly increased and decreased metabolites are indicated using red and blue arrows, respectively. The MCT-specific unique up- and downregulated metabolites are indicated as up- and down-headed brown arrows within white circles while Su/Hx-specific unique downregulated metabolites are indicated as down-headed blue arrows within white circles. MCT, monocarboxylate transporters; GLUT, glucose transporter; FA, fatty acid; FFA, free fatty acid; SLC, solute carrier; NAD, Nicotinamide adenine dinucleotide; NADP+, Nicotinamide adenine dinucleotide phosphate; NADPH, reduced nicotinamide adenine dinucleotide phosphate; ROS,; GSH, glutathione; GSSG, glutathione disulfide; PO4, phosphate; AMP, adenosine monophosphate; IMP, inositol monophosphate; InsP3, inositol triphosphate; OXPHOS, oxidative phosphorylation; ETC, electron transport chain; TCA, tricarboxylic acid cycle; CPT, carnitine palmitoyltransferase; DHAP, Dihydroxyacetone phosphate; GA3P, glyceraldehyde 3-phosphate; α-KG, alpha ketogluterate; Gαq, G-protein coupled receptor alpha-q subunit; PLCβ, phospholipase C beta.

Here we performed the first-ever comprehensive comparative targeted metabolomic analysis on the RV tissue of MCT and Su/Hx rat models of severe decompensated PH-RVF, which revealed distinct model-specific metabolomic signatures with significant overlap of metabolites and metabolic pathways between the two models. Interestingly, our metabolic pathway enrichment analysis showed significant concordance in the RV metabolic reprogramming with “Warburg effect” being the top common pathway in both models. Another unique feature of this study is the JPA using transcriptomic and metabolomic data sets from the RV of MCT and Su/Hx rats demonstrating the correlation between genes and metabolites from critically essential metabolic pathways (Figure 8).

### RV metabolic reprogramming reported in various animal models of PH

Recently, Graham et al. performed a steady-state metabolomics analysis in the RV tissue of female mice (hypoxia or Schistosoma) and female SD rats with hypoxia only or Sugen followed by 3 weeks of hypoxia and 2 (Su-Hx + 2) or 5 (Su-Hx + 5) weeks of normoxia (30). They reported that the RV metabolic substrate delivery was functionally preserved without evidence of depletion of key metabolites. Specifically, there was a significant increase in GSH in Su-Hx + 5 rats, consistent with our findings from RVs of both MCT and Su/Hx rats (Figures 3, 5). Moreover, their data showed metabolic changes including dysregulation of TCA cycle and energy currency metabolites in Su-Hx + 5 rats (decreased α-KG, malate and ADP), consistent with an increased glycolytic shift rather than conventional OXPHOS, which is similar to other studies from the failing heart (27, 29, 37–41). Interestingly, we also found dysregulation of TCA cycle (decreased α-KG and citrate) and significant depletion of critical energy currency metabolites (e.g., creatine and phosphate) in the RVs of both MCT and Su/Hx rats (Figures 3, 5). In addition, our metabolic pathway enrichment analysis found enrichment of arginine and proline metabolism, alanine, aspartate and glutamate metabolism, glutathione metabolism, and citric acid (TCA) cycle, similar to the study of Graham et al. Our results are also in agreement with their study, as no significant changes were detected in lactate and glutamate, although significant decrease in glutamine in both MCT and Su/Hx and significant increase in glucose in MCT rat RVs were observed. However, there are major differences between our study and the study by Graham et al. such as the use of male rats, side-by-side comparison of MCT and Su/Hx rat models of decompensated RVF, and JPA (Figure 7D) in our study. Further, in contrast to their Su-Hx + 5 rat RV metabolomic signature, we found a much higher number of significantly altered metabolites (24 metabolites) and metabolic pathways (60 pathways) in our Su/Hx rats (30). Another difference between our study and the study performed by Graham et al., besides the gender, is the use of hypobaric vs. normobaric hypoxia. Studies have demonstrated that normobaric and hypobaric hypoxia may lead to differential physiological responses. Studies have shown higher heart rate, lower arterial oxygen saturation, decreased physical performance, lower ventilatory response and pH, as well as higher oxidative stress in hypobaric hypoxic condition compared to normobaric hypoxia (42–44). Transcriptomic and metabolic signatures in RV, can potentially be differentially affected after exposure to normobaric and hypobaric hypoxia. However, in this study, our main focus was only to reveal the common and unique metabolic signatures in the decompensated RV of MCT and Su/Hx rats under normobaric conditions.

A recent interesting study, investigating metabolic reprogramming in the RV of murine models of hypoxia alone and hypoxia + Sugen, demonstrated an increase in glutamine, creatine phosphate, lactate and a decrease in free FAs and glucose in both groups compared to normoxic controls. Some of these changes are in contrast with our results, as glutamine and creatine were significantly reduced in our Su/Hx rats, whereas these metabolites were significantly increased in Su/Hx mice used in their study. These findings could be due to fundamental differences between the mouse and rat models of Su/Hx as it is well-established that mice do not demonstrate the evidence of decompensated RVF (31).

Another recent study from Hautbergue et al. investigated the metabolic signatures of RV remodeling in chronic hypoxia- and MCT-treated male Wistar rats and demonstrated significant alterations in metabolites related to arginine, pyrimidine, purine and tryptophan metabolic pathways in the RV of the MCT rats (32). They demonstrated significantly increased thymine, deoxy-uridine and cytosine (pyrimidine metabolic pathway) and significantly decreased creatine and glutamine (arginine metabolic pathway) in MCT RV, which are consistent with our MCT results. Significantly increased tyrosine (tryptophan metabolic pathway) and significantly reduced purine metabolism metabolites such as inosine in their study, are also in concordance with our MCT findings. In addition, we found significantly increased levels of 11 other metabolites including methionine, phosphoethanolamine, glucosamine-6-phosphate, CDP-choline, glucose, valine, proline, phospho-choline, 4-hydroxy phenyllactate, GSH and aspartate in the RV of MCT rats that were not detected in their study. Furthermore, we found significantly decreased levels of 10 other metabolites including carnitine, DHAP, AMP, IMP, α-KG, 5-oxoproline, phosphate, hexose phosphate, pantothenic acid and acetyl carnitine, in the RV of MCT rats that were not detected in their study (32).

In a recent study, Prisco et al. performed a quantitative global metabolomic profiling on RVs of MCT rats and documented higher levels of end products of hexosamine biosynthetic, glycolytic, and pentose phosphate pathways. RVs of MCT rats also showed elevated levels of ceramides, dihydroceramides, hexosylceramides, dicarboxylic fatty acids as well as multiple glutaminolysis-related metabolites and mitochondrial enzymes (45). In another recent study, Prisco et al. also profiled 767 metabolites using global metabolomics approach and demonstrated a distinct metabolic signature in MCT RV metabolism (42). Interestingly, nearly all the acylcarnitines were reduced in the MCT RVs. Dysregulated metabolite signatures demonstrated impaired fatty acid oxidation as well as increased glycolysis and glutaminolysis in the MCT RVs, very similar to our results. Furthermore, integrating proteomic and metabolomic data sets, this study also established glycolysis or gluconeogenesis, tricarboxylic acid cycle, and fatty acid degradation as the most altered metabolic pathways in the MCT RVs (46), very similar to our JPA findings.

### Metabolic shift and warburg phenotype in RV of decompensated PH-RVF: Role of glycolysis, FAO, and OXPHOS

Cardiac hypertrophy-induced structural remodeling results in increased reliance on glucose metabolism with a decrease in FAO. Further, metabolic gene reprogramming in the hypertrophied as well as failing hearts has been well-described as a reversion to a fetal metabolic program. Importantly, these metabolic alterations often precede the hypertrophy-related structural changes in the failing heart and represent stepwise distinct early metabolic reprogramming events, prior to both adaptive as well as maladaptive remodeling. Glycolytic shift from FAO may facilitate ventricular hypertrophy and early adaptation to hemodynamic shear stresses (27, 47). Further, prolonged dependence on glucose utilization likely leads to an ultimate state of energy depletion as cardiac hypertrophy subsequently results in decompensated HF (27, 48–50).

The heart generates ATP from a variety of fuels primarily *via* mitochondrial OXPHOS to maintain the contractile function. The main fuels include fatty acids, lactate, ketones, glucose, pyruvate, and amino acids. Myocardial FAO increases in HF associated with diabetes and obesity, while it decreases in HF associated with hypertension (27). We recently reported that FAO and OXPHOS are the top common down regulated pathways whereas glycolysis is one of the top common upregulated pathways in the transcriptomic data sets from RVs of MCT and Su/Hx rats (24). On the contrary, FAO is demonstrated to be increased in RV of rats with PAB (37). Given that FAO is the major source of energy production in ventricular cardiomyocytes (41), lipid metabolism has been understudied in the failing RV in PH. Increased circulating levels of free FAs and increased RV-specific deposition of long-chain FAs, ceramides and triglycerides were documented in the patients with PAH (51–53). Remarkably, Randle cycle describes the reciprocal relationship between the activation of FAO and glucose oxidation (54). Similarly, in our study, we found a significant reduction in acetyl-carnitine in both MCT and Su/Hx rat RVs (Figures 3, 5) and carnitine in MCT rat RVs which clearly suggests significantly reduced transfer of long-chain FAs across the inner mitochondrial membrane for subsequent β-oxidation as mentioned elsewhere (55–57). Further, simultaneous significantly increased levels of glucose in RVs of MCT rats support increased glucose oxidation, because of the reciprocal relationship between FAO and glucose oxidation (Figure 4A). Glycolysis converts glucose to pyruvate which subsequently either converts to lactate or undergoes further mitochondrial oxidation. A shift from mitochondrial FAO to glycolysis occurs in the RV and foundational investigations demonstrated increased glycolysis and reduced glucose oxidation in RV, similar to the “Warburg effect,” well-documented in cancer literature (58, 59). In fact, the metabolic reprogramming in the failing RV and a chronic shift in energy production from OXPHOS to glycolysis (Warburg effect) is associated with PH-induced decompensated RVF (60, 61) and is strongly supported by our data (Figures 7A–C).

Although majority of the studies suggest that diminished FAO and OXPHOS drive metabolic derangement in the RV of PH-RVF, however contemporary reports highlight the involvement of increased glucose oxidation in mediating PH-RVF progression (60, 61). Interestingly, our results are consistent with finding Warburg effect, glycolysis/gluconeogenesis, glutaminolysis, and amino acid metabolism as the top common pathways (Figures 7A–C) and alteration of related metabolites (aspartic acid, glutamine, 5-oxoproline and hexose phosphate). However, the events of metabolic reprogramming during decompensated RVF beyond the conventional “Warburg effect” create more complexity in metabolic rewiring to promote PH-RVF and are becoming the focus of future research (60, 61). Finally, parallels have been drawn between significant alterations in metabolic pathways such as fatty acid oxidation and synthesis, pentose phosphate pathway and glutaminolysis in cancer and PH-induced RV remodeling (61).

Under normoxic conditions, pyruvate is oxidized in the mitochondria by the Krebs cycle to generate ATP and meet energy demands in cells. However, during hypoxic conditions, cells with “Warburg phenotype” utilize pyruvate to produce lactate through the action of lactate dehydrogenase (LDH/LDHA). Warburg Effect or “aerobic glycolysis” can be defined as lactate production in the presence of oxygen. Out of several signaling pathways contributing to the Warburg Effect, receptor tyrosine kinases (RTK)-mediated activation of PI3K/Akt and Ras plays a significant role. Akt is known to stimulate glycolysis through activation of several glycolytic enzymes including hexokinase and phosphofructokinase. RTK signaling results in transcriptional activation of several genes of glycolysis and lactate production. Hypoxia-signaling can increase expression of LDHA to stimulate lactate production, as well as pyruvate dehydrogenase kinase to inhibit pyruvate dehydrogenase and restrict pyruvate entry into the Krebs cycle (58, 62–71).

In our transcriptomic data, very similar to the established “Warburg phenotype” signaling and metabolic signatures, we also found significantly upregulated/increased expression of phosphofructokinase (pfk) and hexokinase3 (hk3) in the RV of both MCT and SuHx rats. Further, LDHA and pyruvate dehydrogenase kinase3 (pdk3) are also significantly increased in the RV of these two groups. Significantly increased expression of Akt (Akt3), tyrosine kinase (Tyk2) and activation of Ras signaling (increased Rassf1) were also documented in the RV of MCT and Su/Hx rats (Supplementary Tables 1, 2: Warburg signaling MCT vs. Ctrl and SuHx vs. Ctrl). Taken together, increased expression of Warburg Effect-related signaling strongly suggests that the decompensated RV of these two groups indeed attains “Warburg phenotype” to support increased pyruvate to lactate conversion and utilization, through “aerobic glycolysis” due to the metabolic shift.

Further, secondary reactome analysis on the transcriptomic data from the decompensated RV of MCT and Su/Hx rats, confirmed that pathways related to free fatty acid (FFA) metabolism such as fatty acid metabolism, mitochondrial fatty acid ß-oxidation, ß-oxidation of saturated fatty acids, and import of palmitoyl-CoA into the mitochondrial matrix were significantly decreased in the RV of these two severe models of PH-induced RVF. Further, ß-oxidation of deconoyl-CoA to octanoyl-CoA CoA was also found to be decreased in the RV of Su/Hx rats. Hence, reactome analysis clearly confirmed decreased FFA metabolism in the RV of both MCT and Su/Hx rats (Supplementary Table 3: Downregulated FFA metabolism).

### Metabolic reprogramming in RV of decompensated PH-RVF: Role of glutaminolysis

In addition to alterations in FAO, glycolytic shift, glucose oxidation, and OXPHOS, amino acid metabolism particularly glutaminolysis has also been documented in the decompensated RVs of our MCT and Su/Hx rats similar to other previous studies (72–75). During the stepwise metabolic reprogramming while transitioning from adaptive to maladaptive RV remodeling, in addition to the “Warburg phenotype,” increased utilization of glutamine through glutaminolysis to replenish the carbon intermediates in the TCA cycle is also documented (27, 28). Glutaminolysis is an anaplerotic deamination reaction that converts glutamine to glutamate by glutaminase and subsequently glutamate to α-KG *via* glutamate dehydrogenase. TCA intermediates, derived from glutaminolysis, especially α-KG participate in FA, amino acid, and *de novo* purine as well as pyrimidine biosynthesis (76). Glutaminolysis is an alternative upregulated metabolic pathway, associated with Warburg phenotype, documented in cancer studies. MCT-induced decompensated RV remodeling demonstrated increased glutaminolysis and increased expression of glutamine transporters, which was not seen in the more adaptive PAB model (29). Glutamine transporters are also upregulated in the RV of patients with PAH-RVF (41) consistent with metabolic remodeling (26).

### Metabolic reprogramming in RV of Decompensated PH-RVF: Role of TCA cycle

In the current study, we also identified tricarboxylic acid (TCA) cycle (citric acid cycle) as one of the top common dysregulated metabolic pathways in RV of MCT and Su/Hx rats (Figure 7). α-ketoglutarate (α-KG), as a crucial intermediate metabolite of TCA cycle, regulates ATP production and mitochondrial energy homeostasis (77–79). Interestingly we found a significant decrease in α-KG in both MCT and Su/Hx rats (Figure 3C). α-KG supplementation has been shown to improve cardiac contractile dysfunction in transverse aortic constriction (TAC) mice by attenuating pressure overload-induced cardiac hypertrophy and fibrosis. Further, α-KG exerts cardioprotective effects in TAC-induced failing myocardium by: (1) reducing ROS production and oxidative stress, (2) inhibiting cardiomyocyte apoptosis, (3) promoting autophagy and mitophagy, and (4) improving mitochondrial membrane potential (79, 80).

### Metabolic reprogramming in RV of decompensated PH-RVF: Role of inositol phosphate metabolism

Inositol 1,4,5-trisphosphate (IP3) is a crucial intracellular second messenger regulating diverse cardiac functions, including pacemaking, excitation–contraction as well as excitation-transcription coupling to the initiation as well as progression of ventricular hypertrophy and arrhythmias. Furthermore, strategic cytoplasmic and nuclear compartmentalized localization of IP3-receptors (IP3R) allows them to participate in subsarcolemmal, cytoplasmic as well as nuclear Ca^2+^ signaling in ventricular cardiomyocytes. IP3R expression levels are shown to be increased in hypertension, cardiomyocyte hypertrophy, failing myocardium as well as ischemic dilated cardiomyopathy (81–88). Similarly, our RV transcriptomic data showed significantly increased IP3R3 and IP3R-interacting protein mRNA transcripts in MCT rats and only the ITPRIP Like 2 mRNA transcripts in Su/Hx rats (Supplementary Table 4, Inositol-metabolism-signaling). Inositol-polyphosphate 5-phosphatases (Inpp5) and inositol polyphosphate-4-phosphatases (Inpp4) are enzymes catalyzing the conversion of inositol triphosphates to bisphosphates and may be involved in regulating cardiomyocyte hypertrophy and contractility. Specifically, inositol polyphosphate-5-phosphatase 5f (Inpp5f) is reported as a key player in regulating cardiac hypertrophic responsiveness (89). Our transcriptomic data also revealed significant elevation in all the inositol polyphosphate-5-phosphatases in the RV of both these groups (Supplementary Table 4, Inositol-metabolism-signaling). However, significantly decreased mRNA transcripts of inositol monophosphatase a (Impa) 1/2 and Inpp4b were documented in MCT and Su/Hx rats (Supplementary Table 4, Inositol-metabolism-signaling). The genes involved in complex IP3/IP3R signaling as well as inositol metabolism, metabolic intermediates and derivatives showed very similar signature in the RV of both MCT and SuHx rats.

Our metabolomics data demonstrated significantly reduced inositol in RVs of both MCT and Su/Hx rats and inositol monophosphate (IMP) in the MCT group (Figures 3–5). Similarly, a recent study demonstrated reduced levels of myo-inositol in the RV myocardium of rabbits with myocardial infarction (90). Further, oral supplementation of myo-and d-chiro-inositol rescues cardiac function and remodeling in diet-induced obese mice (91). We postulate that during the decompensatory stage, hypoxic and hypertrophied RV cardiomyocytes may utilize the pool of inositol and IMP to combat hypoxia-induced stress. These hypoxic and hypertrophied cardiomyocytes may also channelize inositol and IMP to balance Ca^2+^/IP3 dynamics and maintain their contractile function.

### Metabolic reprogramming in RV of decompensated PH-RVF: Role of amino acid metabolism

Metabolomic analysis from other studies have revealed increased flux into non-oxidative pathways especially amino acid metabolism, which is well-documented in hypertrophic and failing hearts (27, 29, 92–94). Further, majority of the significantly altered amino acids are involved in the TCA cycle, nucleotide metabolism as well as arginine/urea cycle as metabolic intermediates (27, 29, 40, 92, 93, 95). Interestingly, we found significantly increased aspartic acid in both models. Further, we found increased methionine, valine, proline, and tyrosine in MCT rats and decreased valine, leucine/isoleucine, lysine, arginine, histidine and asparagine in Su/Hx (Figures 3–6). Although amino acids offer minor contribution to overall cardiac OXPHOS due to their low availability under normal conditions (94), branched-chain amino acids (BCAAs) oxidation is considered as a major source of ATP production in the heart (96). Of note, we found significantly decreased BCAAs such as valine and leucine/isoleucine in Su/Hx rats.

### Limitations

As a limitation of this study, we did not investigate metabolomic changes in RV of compensated RVH, or RVF secondary to pure RV pressure overload such as PAB or in female rats. We also did not investigate LV metabolomics in rats with decompensated RVF. Additionally, we performed targeted analysis of a set of 150 central carbon metabolites and detected ~125 metabolites as not all metabolites were detectable in all samples, depending on the abundance of the metabolites and/or matrix effects. As a result, we may have missed some key metabolites linked to pathways such as fatty acid oxidation. Furthermore, although the metabolomic signatures in our study most likely represent the metabolites predominantly found in cardiomyocytes, however recent studies have highlighted the contribution of other cardiac cell types and non-cardiomyocyte populations, especially fibroblasts (97). Future studies highlighting single cell metabolomic signatures are certainly warranted.

## Conclusions

In conclusion, in the current study, unbiased metabolic profiling provided a comparative and comprehensive understanding of metabolic reprogramming that occurs in the RV of two severe rat models of decompensated RVF and resulted in the discovery of previously unappreciated biological pathways that contribute to PH-RVF pathogenesis (Figures 7, 8). Further, comparative analysis of metabolic reprogramming of RV revealed common and distinct metabolic signatures from MCT and Su/Hx PH-RVF rats. These metabolic signatures may serve as novel, targeted and effective therapeutic targets for PH-RVF.

## Data availability statement

The raw data supporting the conclusions of this article will be made available by the authors, without undue reservation.

## Ethics statement

The animal study was reviewed and approved by University of California Los Angeles Animal Research Committee (ARC).

## Author contributions

SB, JH, and SU were responsible for collecting, analyzing, and interpreting the data. SB and SU wrote the manuscript. SU supervised the study. All authors contributed to the article and approved the submitted version.

## Funding

This work was supported by the NIH NHLBI Grant1K08HL141995 (SU).

## Conflict of interest

The authors declare that the research was conducted in the absence of any commercial or financial relationships that could be construed as a potential conflict of interest.

## Publisher's note

All claims expressed in this article are solely those of the authors and do not necessarily represent those of their affiliated organizations, or those of the publisher, the editors and the reviewers. Any product that may be evaluated in this article, or claim that may be made by its manufacturer, is not guaranteed or endorsed by the publisher.
