# Supplementary figures and images for: Comparative analysis of right ventricular metabolic reprogramming in pre-clinical rat models of severe pulmonary hypertension-induced right ventricular failure
Source: Front Cardiovasc Med. 2022 Sep 9;9:935423. doi: 10.3389/fcvm.2022.935423 (PMC9500217; doi:10.3389/fcvm.2022.935423)

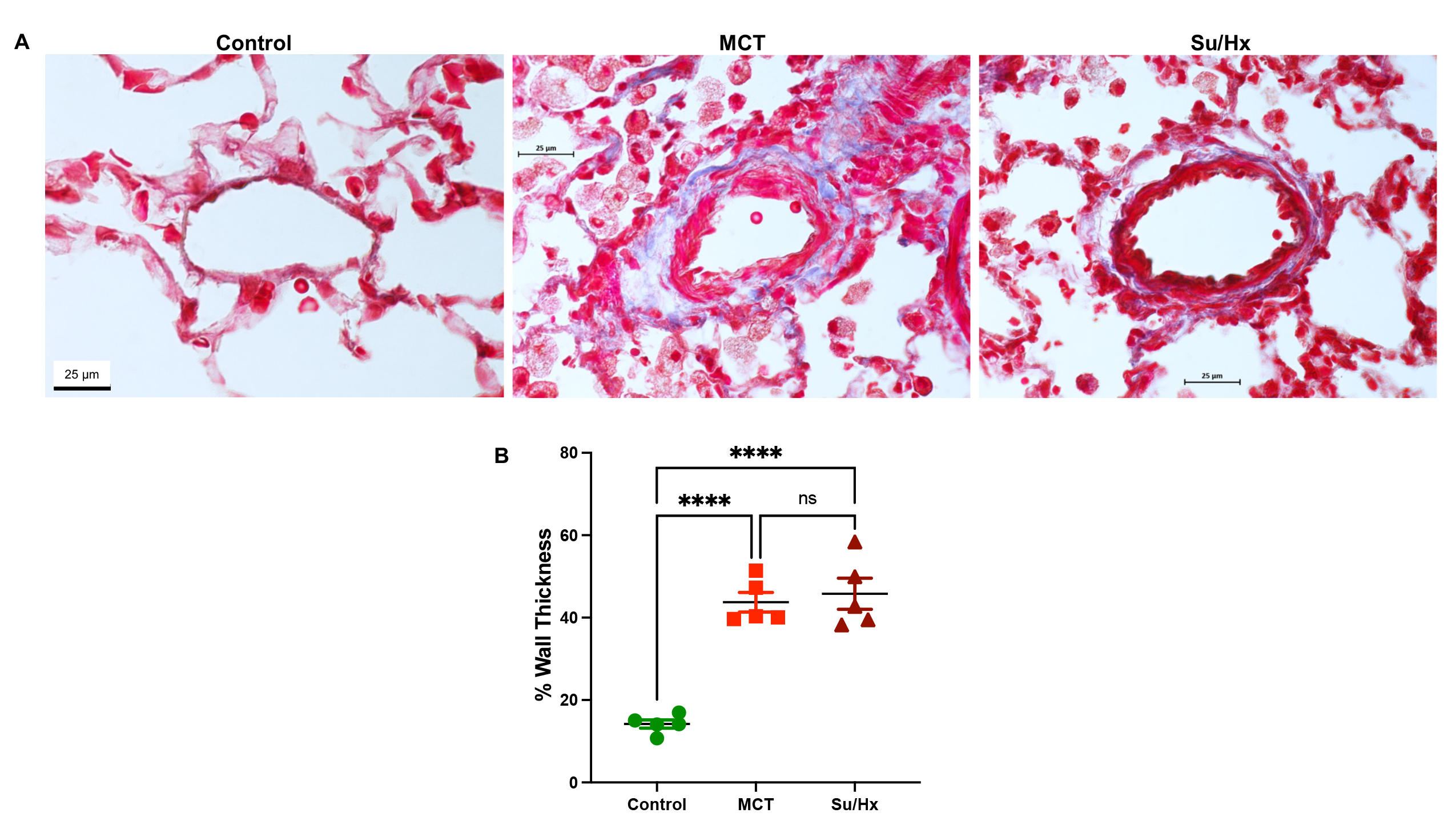

Supplement: Supplementary file 1 [file Image_1.TIFF]

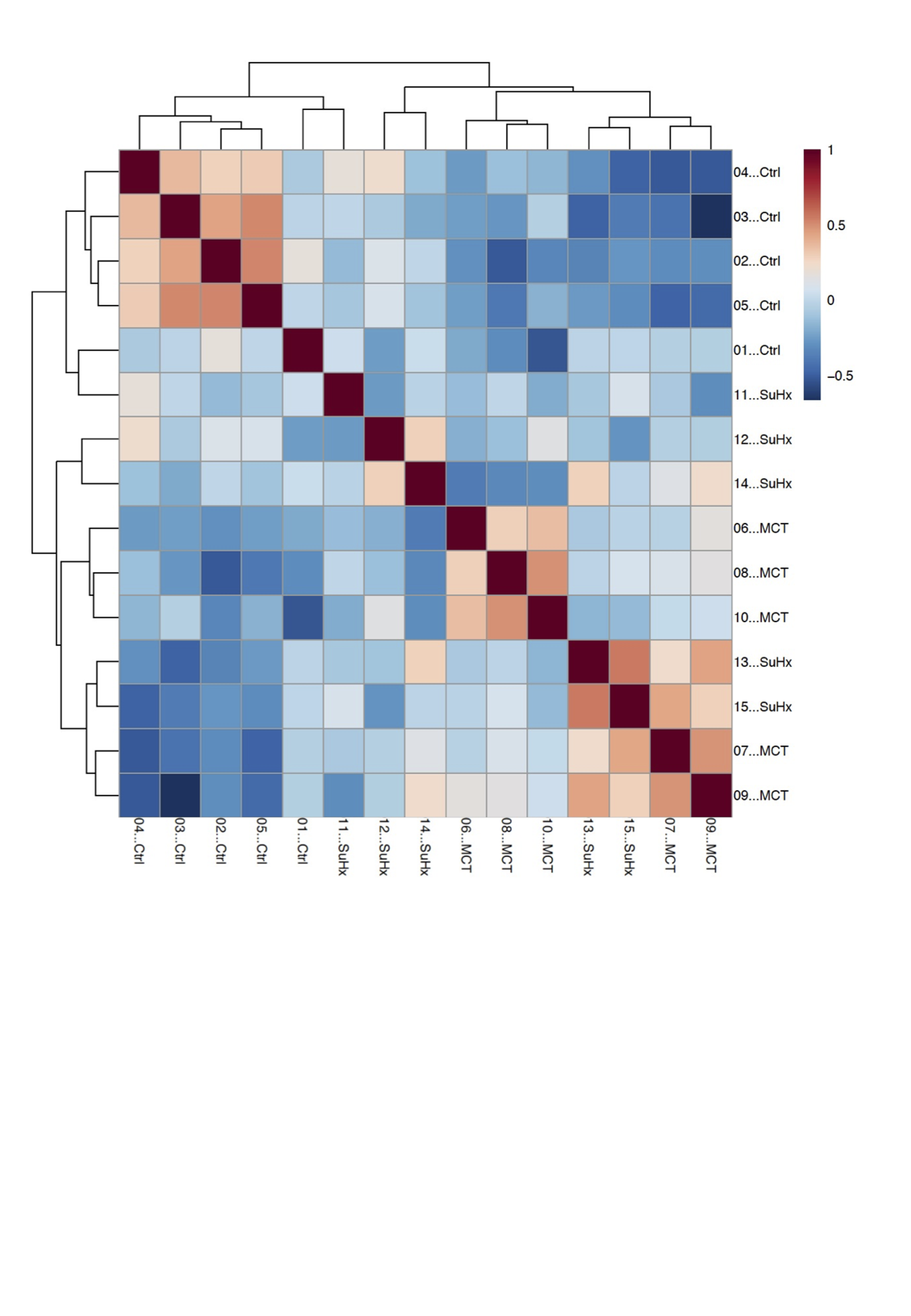

Supplement: Supplementary file 2 [file Image_2.TIFF]

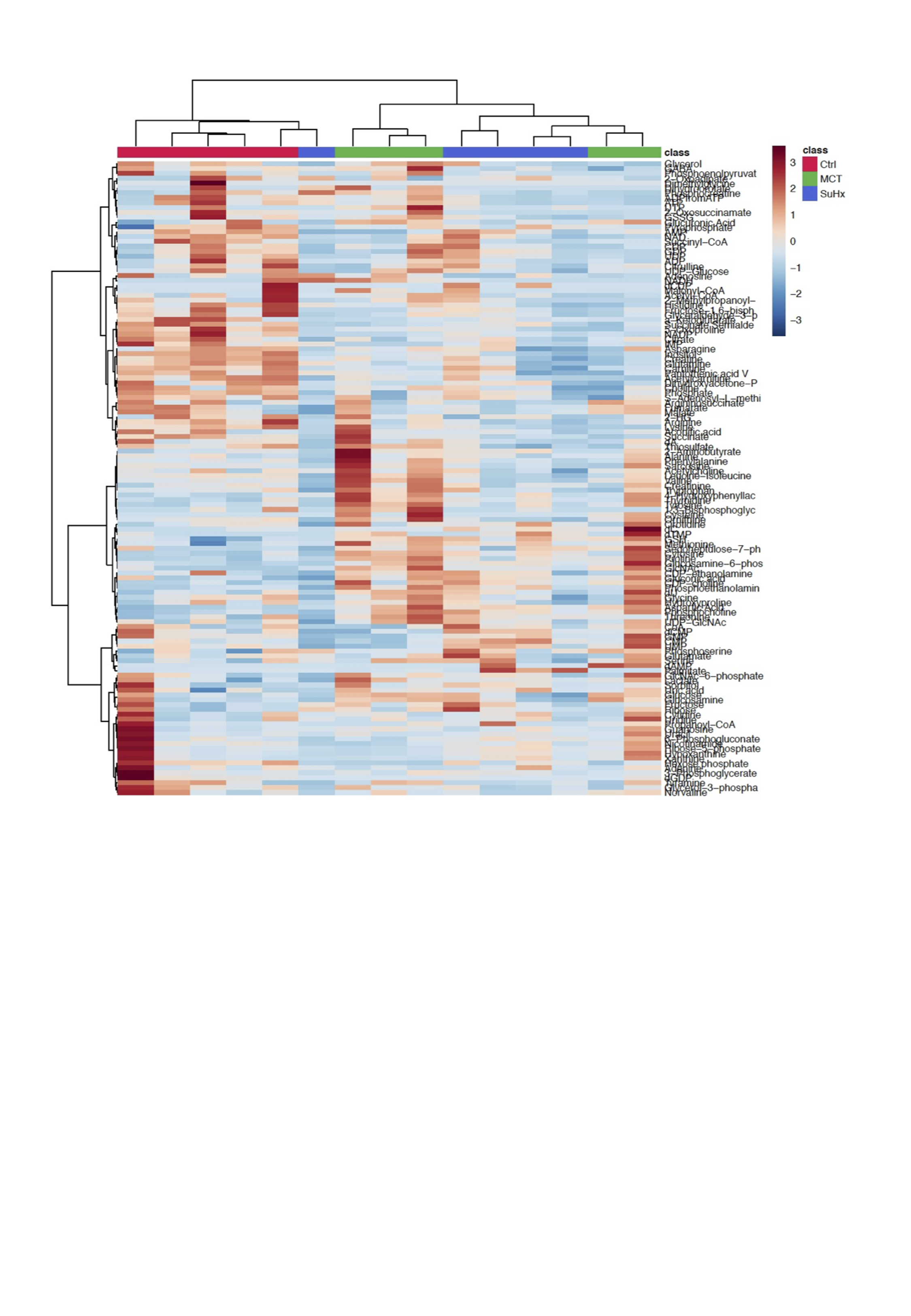

Supplement: Supplementary file 3 [file Image_3.TIFF]

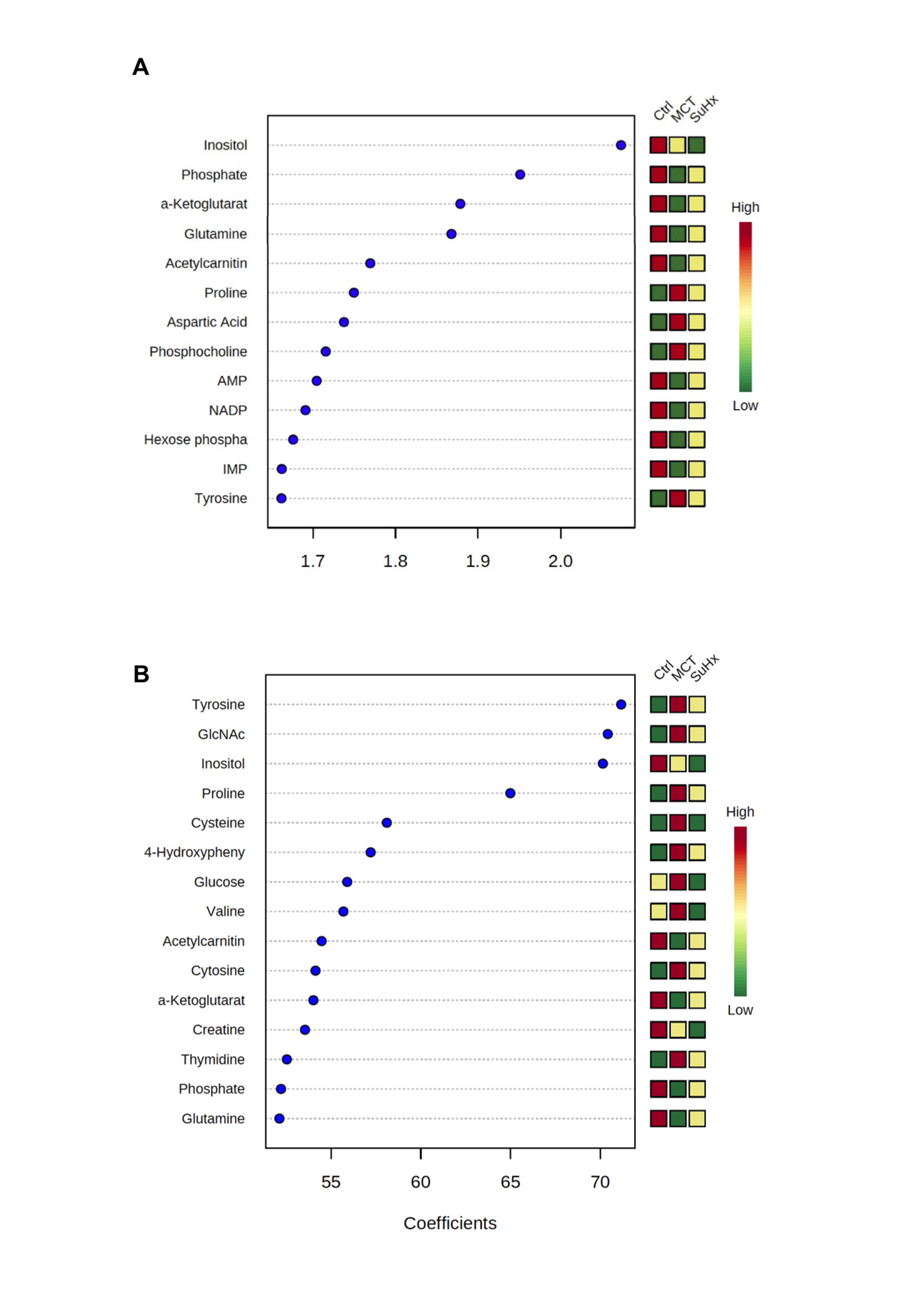

Supplement: Supplementary file 4 [file Image_4.TIFF]

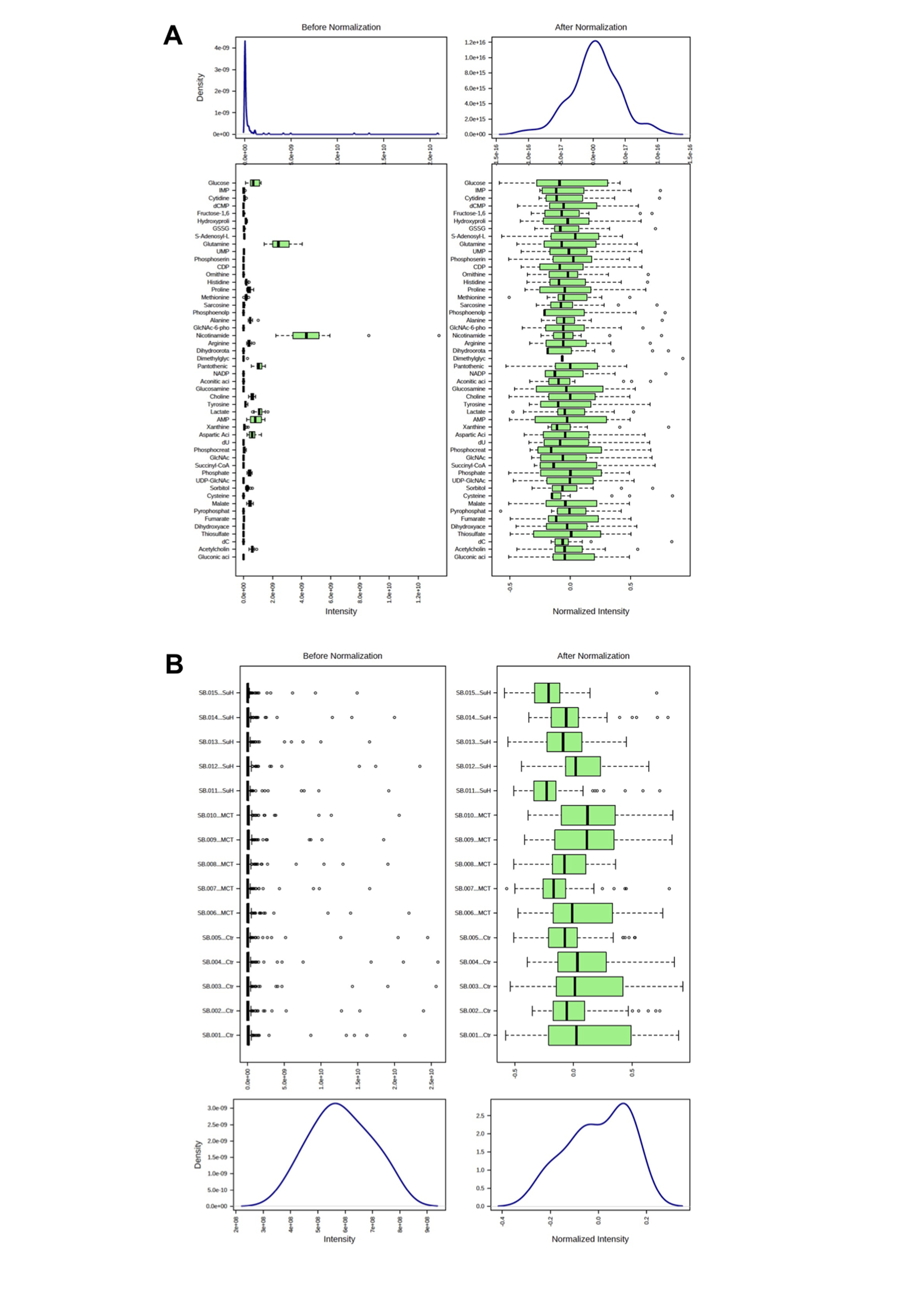

Supplement: Supplementary file 5 [file Image_5.TIFF]

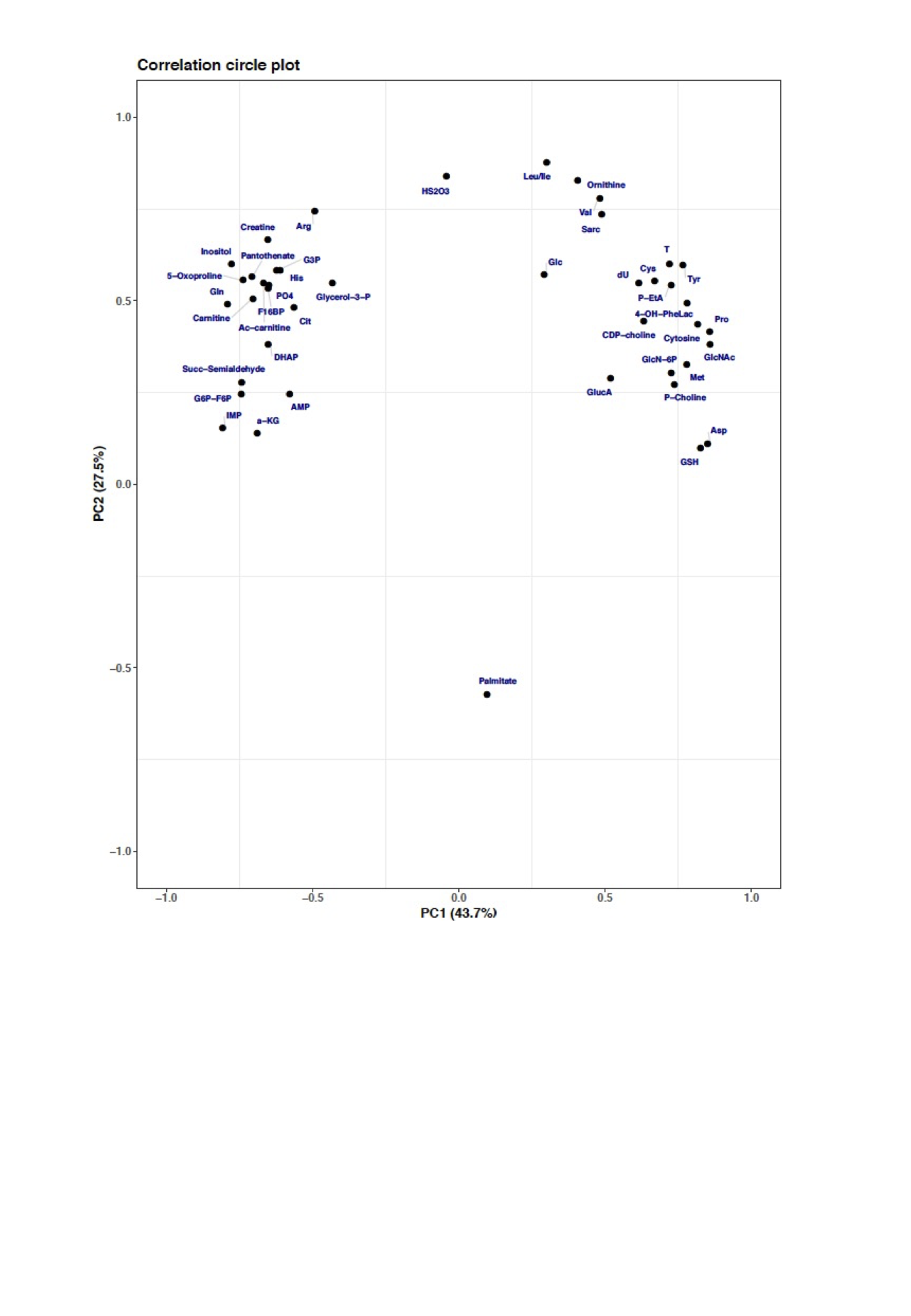

Supplement: Supplementary file 6 [file Image_6.TIFF]

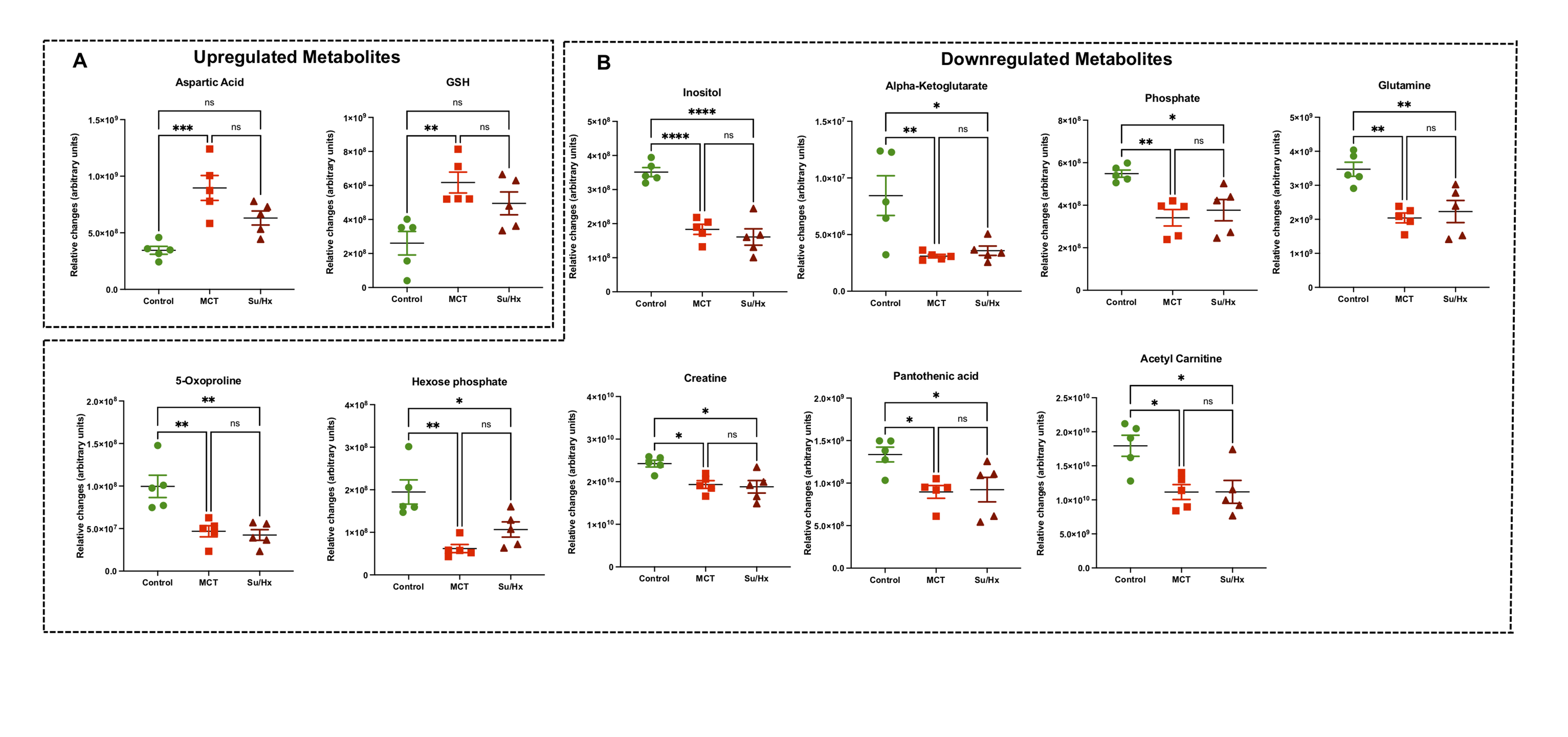

Supplement: Supplementary file 7 [file Image_7.TIFF]

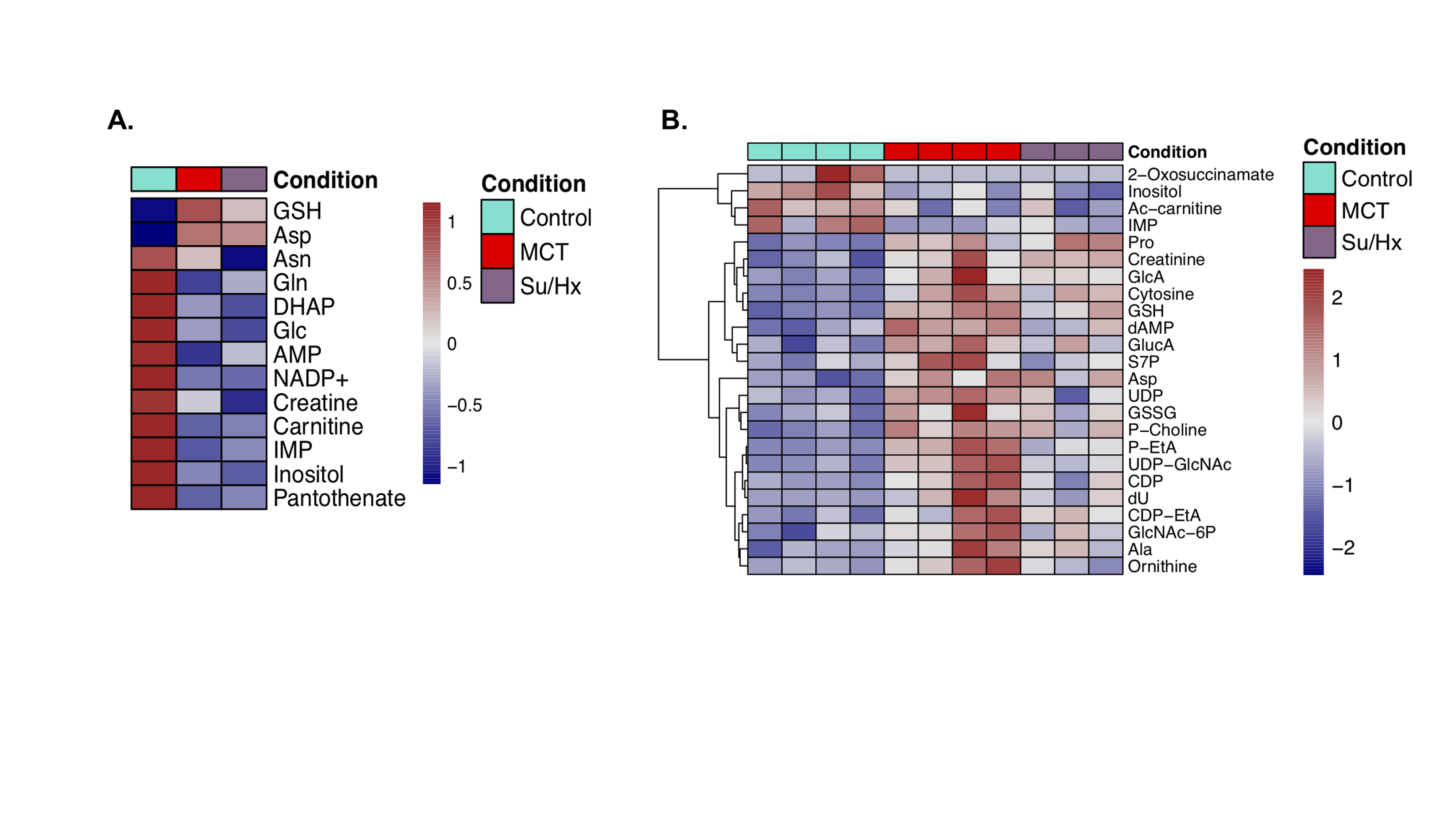

Supplement: Supplementary file 8 [file Image_8.TIFF]
