# Supplementary material for: Comparative analysis of right ventricular metabolic reprogramming in pre-clinical rat models of severe pulmonary hypertension-induced right ventricular failure
Source: Front Cardiovasc Med. 2022 Sep 9;9:935423. doi: 10.3389/fcvm.2022.935423 (PMC9500217; doi:10.3389/fcvm.2022.935423)
Supplement: Supplementary file 9 [file Data_Sheet_1.pdf]

SUPPLEMENTAL MATERIAL

I. Supplementary Figure Legends

Figure S1. Masson's trichrome staining of lung sections showing pulmonary vascular remodeling (**A**) and quantification of percentage wall thickness (**B**) showing severe pulmonary vascular remodeling documented as significantly increased pulmonary arteriolar medial hypertrophy in MCT and Su/Hx rats compared to control. N=5 per group. Data represented as mean ± SEM. ****p<0.0001.

Figure S2. Correlation heat map of individual rats from Control, MCT and Su/Hx groups and their hierarchcial clustering based on targeted metabolomics data from RV tissue. Red color represents positive correlation and blue color represents negative correlation.

Figure S3. Heat map of scaled expression of metabolites from RV samples of Control (red), MCT (green) and Su/Hx (blue) rats. Red color represents high expression and blue color represents low expression. N=5 per group.

Figure S4. VIP scores and coefficients from targeted metabolomics data from RV of Ctrl, MCT and Su/Hx rats as determined by Partial-Least Squares Discriminant Analysis in MetaboAnalyst. VIP, Variable Importance in Projection. Red color represents high relative concentration and green color represents low relative concentration.

Figure S5. Normalization plot from targeted metabolomics data of RV tissue from Ctrl, MCT and Su/Hx rats.

Figure S6. Correlation circle plot from targeted metabolomics data of RV tissue from Ctrl, MCT and Su/Hx rats showing individual metabolite correlations with the first and second principle components.

Figure S7. Comparative analysis of targeted metabolomics of RV tissue from severe decompensated RV Failure of MCT and Su/Hx rats compared to Controls highlighting common metabolites. **A.** Upregulated metabolites from RV tissue of MCT (red) and Su/Hx (brown) compared to Control rats (green). **B.** Downregulated metabolites from RV tissue of MCT (red) and Su/Hx (brown) compared to Control rats (green). Data presented as mean±SEM. N=5 per group. *p<0.05, **p<0.01, ***p<0.001, ****p<0.0001.

Figure S8. Comparative analysis of targeted metabolomics of RV tissue from severe decompensated RV Failure in validation cohort of MCT and Su/Hx rats compared to Controls. **A.** Condensed, averaged heat map of 13 top metabolites from targeted metabolomics data of RV tissue from the validation cohort of Control (green; N=4), MCT (red; N=4) and Su/Hx (purple; N=3) rats. Red color represents upregulated and blue color represents downregulated metabolites. **B.** Heat map showing scaled expression of 24 differentially expressed metabolites from RV tissue of Control (green; N=4), MCT (red; N=4) and Su/Hx (purple; N=3) rats. Metabolites are represented with their

normalized average amounts [upregulated (red) and downregulated (blue); (FDR<0.05)].

II. Supplementary Tables

Supplementary Table 1. List of differentially expressed Warburg signaling and metabolic genes from the RV of MCT rats. List of differentially expressed Warburg signaling and metabolic genes that were found statistically significant in the RV of MCT rats compared to Controls. Data is annotated to include gene symbol, gene description, ensemble ID, log2fold change, lfcSE (log2fold change standard error), p-value, and corrected p-value (Benjamini-Hochberg).

Supplementary Table 2. List of differentially expressed Warburg signaling and metabolic genes from the RV of Su/Hx rats. List of differentially expressed Warburg signaling and metabolic genes that were found statistically significant in the RV of Su/Hx rats compared to Controls. Data is annotated to include gene symbol, gene description, ensemble ID, log2fold change, lfcSE (log2fold change standard error), p-value, and corrected p-value (Benjamini-Hochberg).

Supplementary Table 3. List of down-regulated pathways associated with Free Fatty acid (FFA) metabolism from the RV of MCT and Su/Hx rats. List of down-regulated pathways associated with Free Fatty acid (FFA) metabolism in the RV of MCT and Su/Hx rats compared to Controls. The data is presented as category (metabolism), reactome pathway, adjusted p-value and normalized enrichment of term (NES).

Supplementary Table 4. List of differentially expressed genes involved in Inositol metabolism and signaling in the RV of MCT and Su/Hx rats. List of differentially expressed genes involved in Inositol metabolism and signaling that were found statistically significant in the RV of MCT and Su/Hx rats compared to Controls. Data is annotated to include gene symbol, gene description, ensemble ID, log2fold change, lfcSE (log2fold change standard error), p-value, and corrected p-value (Benjamini-Hochberg).
